# Cómo manejar las muestras lipémicas para realización de contaje de células sanguíneas (CBC) en los analizadores hematológicos Sysmex

**DOI:** 10.1515/almed-2025-0037

**Published:** 2025-04-01

**Authors:** Vanja Radišić Biljak, Lucija Dolovčak, Iva Bakarić, Ana Nikler, Andrea Saračević, Marija Grdić Rajković

**Affiliations:** Department of Medical Laboratory Diagnostics, EuSpLM, University Hospital „Sveti Duh“, Sveti Duh 64. 10000, Zagreb, Croatia; Servicio de Diagnóstico del Laboratorio Clínico, Hospital Universitario Sveti Duh de Zagreb, Zagreb, Croatia; Departamento de Medicina del Deporte y el Ejercicio Físico de la Universidad de Zagreb, Facultad de Kinesiología, Zagreb, Croatia; Departamento de Bioquímica Clínica y Hematología de la Facultad de Farmacia y Bioquímica de la Universidad de Zagreb, Zagreb, Croatia

**Keywords:** hemograma completo (CBC), hemoglobina, lipemia, interferencia

## Abstract

**Objetivos:**

La lipemia es un problema preanalítico reseñable para el proceso de recuento de células sanguíneas (CBC), ya que no existe un método estandarizado para su identificación y eliminación. El objetivo de presente estudio es verificar la medida de la hemoglobina óptica (Hb-O) en el hemoanalizador (HA) Sysmex XN-1000 como método fiable para el manejo del CBC en muestras lipémicas.

**Métodos:**

Empleando una emulsión de lípidos, se procedió a enriquecer gradualmente 90 muestras de CBC con concentraciones diferentes de Hb. Las determinaciones se repitieron y se registraron las concentraciones de Hb-O. Las muestras de CBC enriquecidas se centrifugaron (400 *g*/10 minutos). A continuación, se extrajo cuidadosamente el plasma y se midió la concentración de Hb. Los valores obtenidos de las muestras lipémicas se ajustaron de acuerdo a las determinaciones en plasma. El plasma eliminado se sustituyó con el diluyente del analizador, y se repitieron las mediciones. Se midieron las concentraciones de triglicéridos en el plasma de muestras lipémicas.

**Resultados:**

Se observó un sesgo estadísticamente no significativo y aceptable en la Hb-O con respecto a la determinación inicial de Hb, según los requisitos más estrictos de aceptabilidad (−0,4 %, IC95 %:−1,2–0,3, p=02,447). El sesgo observado no se correlacionó con el grado de lipemia (rho=−0,072, IC95 %:−0,295–0,157, p=0,537). La hemoglobina medida en muestras de plasma lipémico sustituidas por un diluyente del analizador mostró un sesgó mínimo, aunque estadísticamente significativo (−1,1 %, IC95 %:−2,0–(−0,1), p=0,025). El sesgo observado fue inversamente proporcional al grado de lipemia (rho=−0,369, CI 95 %:−0,550 – (−0,155), p=0,001). El mayor sesgo inaceptable se obtuvo al recalcular los valores de hemoglobina según la hemoglobina medida en plasma (−3,5 %, IC95 %: −4,1 – (−2,9), p<00,001).

**Conclusiones:**

La determinación de Hb-O es el método más fiable a la hora de eliminar la lipemia en muestras para recuento CBC en el hemoanalizador Sysmex XN-1000.

## Introducción

La lipemia, definida como la turbidez de la muestra debido a la acumulación de partículas de lipoproteínas, es un problema importante en el laboratorio clínico [1]. Aunque es relativamente poco frecuente, es una dificultad preanalítica importante para la realización del hemograma completo en muestras lipémicas de sangre total por diversas razones. Dado que la mayoría de los analizadores de hematología (AH) miden la hemoglobina mediante espectrofotometría a 530 nm, la lipemia interfiere con la determinación de la hemoglobina, dando lugar a concentraciones falsamente elevadas de hemoglobina y, en consecuencia, una hemoglobina corpuscular media (MCH) y concentraciones de hemoglobina corpuscular media (MCHC) falsamente elevadas [2]. En los análisis de bioquímica y endocrinología, las interferencias por hemólisis, ictericia y lipemia se suelen identificar mediante inspección visual, mediante determinaciones HIL (semi)cuantitativas, o por una combinación de ambas [3]. Por el contrario, la alerta más común que indicaría una muestra de sangre lipémica en el recuento hematológico (CBC) es el aumento de MCHC por encima de un umbral determinado. Aunque algunos autores proponen un punto de corte de 360 g/L para el MCHC para indicar la presencia de lipemia [4], cada laboratorio debe establecer su propio límite para la lipemia, por encima del cual los resultados no se deben ser informados [5]. Los laboratorios de hematología de todo el mundo cuentan con diversos métodos para eliminar la interferencia por lipemia en el conteo sanguíneo completo [6]. Uno de estos métodos empleados es la determinación de la concentración de hemoglobina en muestras de plasma lipémicas centrifugadas. El valor de hemoglobina obtenido en muestras de plasma lipémicas permite una mejor corrección de la concentración de hemoglobina en sangre total. A la concentración de hemoglobina de la muestra de sangre total se le resta la concentración de hemoglobina medida en la muestra de plasma lipémica. A continuación, tras la corrección, se recalculan los valores de MCH y MCHC. Otro procedimiento muy común para eliminar la lipemia es el procedimiento de sustitución de plasma. Dicho procedimiento consiste en eliminar el plasma lipémico y sustituirlo por el mismo volumen de diluyente del analizador, tras lo cual, se vuelve a analizar la muestra. La solución más práctica a la hora de obtener resultados de CBC fiables de las muestras de sangre total lipémicas es emplear analizadores de hematología insensibles a la interferencia por lipemia. El analizador Siemens Advia 2120i cuantifica la concentración de hemoglobina corpuscular media (CHCM) celular midiendo directamente la hemoglobina en los eritrocitos intactos. Este método permite determinar con precisión la hemoglobina sin la interferencia por lipemia [7], 8], 9].

En 2019, el Grupo de Trabajo Croata de Hematología de Laboratorio de la Sociedad Croata de Bioquímica Clínica y Medicina de Laboratorio, quiso explorar la práctica habitual de hematología de laboratorio en los laboratorios croatas, con el fin de desarrollar futuras estrategias para la elaboración de recomendaciones nacionales que promuevan la armonización en este campo [5]. La encuesta realizada reveló el uso de diversas estrategias para resolver las interferencias por lipemia en el CBC [5]. La estrategia más común es determinar la concentración de hemoglobina en muestras de plasma lipémicas centrifugadas, que representaban el 40 % de los casos [5]. Sin embargo, dicho método implica una manipulación considerable de la muestra, precisando recalcular los resultados, para garantizar la obtención de unos valores de hemoglobina fiables.

Hasta hace poco, en nuestro laboratorio se utilizaban los analizadores de hematología Siemens Advia 2120i, por lo que la lipemia no suponía ningún problema para la realización de los hemogramas. Posteriormente, estos analizadores fueron sustituidos por los analizadores Sysmex XN-1000. Con el fin de optimizar y estandarizar nuestra práctica, así como garantizar la seguridad del paciente, realizamos un estudio para establecer un protocolo local de interferencia por lipemia para garantizar determinaciones de hemoglobina fiables. Aunque el analizador Sysmex XN-1000 reconoce esta posible interferencia de la muestra, el fabricante no ofrece ninguna solución práctica para la eliminación de la lipemia en la sección de limitaciones del sistema del manual de instrucciones del analizador de hematología automatizado XN series (XN-1000). A raíz de los resultados publicados por Berda-Haddad y col. [10], Sysmex propuso un algoritmo para resolver las determinaciones de MCHC falsamente elevadas. Dicho algoritmo se puede integrar en el *extended IPU* del laboratorio (EPU), en caso de que se disponga del mismo. Según el algoritmo propuesto por Sysmex, la elevada turbidez de la muestra es una de las posibles causas de la obtención de valores elevados de MCHC, siendo la determinación de hemoglobina óptica en el modo de cuantificación de reticulocitos (Hb-O) un método fiable para eliminar la interferencia por lipemia en las muestras de sangre [11]. Sin embargo, actualmente, la Hb-O solo se utiliza en contextos experimentales y no está incluida en el menú de control de calidad (QC). Por todo ello, evaluamos las características analíticas del nuevo modo que permite la determinación de la hemoglobina óptica y las comparamos con los protocolos de eliminación de lipemia más comunes.

## Materiales y métodos

El estudio experimental se realizó en el Servicio de Diagnóstico del Laboratorio Cínico del Hospital Universitario Sveti Duh de Zagreb en Croacia, entre octubre y noviembre de 2024.

### Precisión

Previamente a evaluar la lipemia, realizamos un breve estudio de precisión de las determinaciones de Hb-O en el analizador de hematología Sysmex XN-1000 (HA) (Sysmex, Kobe, Japón). Analizamos la precisión intradía en muestras de pacientes con tres intervalos de concentración diferentes, así como en muestras de control comerciales (Sysmex XN Check, lote:Nivel 1 42461101, Nivel 2 42461102, Nivel 3 42461103, exp:24.11.2024.) realizando 20 análisis consecutivos. La precisión interdía se determinó cada día durante treinta días, empleando muestras de control comerciales. Se calculó el coeficiente de variación para realizar el estudio de precisión intra- e interdía.

### Exactitud

En el HA Sysmex XN-1000, se analizaron las solicitudes de hemograma completo de 90 muestras de sangre total K_2_EDTA de pacientes con diferentes concentraciones de Hb, El resultado del hemograma inicial se consideró el valor real. Para garantizar la diversidad de concentraciones de Hb, las muestras se seleccionaron según la distribución presentada en la Tabla 1.

**Tabla 1:** Distribución de las muestras de sangre total con K_2_EDTA con el protocolo de eliminación de lipemia.

Concentración inicial de Hb, g/L	Adición de la emulsión lipídica, µL					
	20	40	60	80	100	120
50–110	5	5	5	5	5	5
110–150	5	5	5	5	5	5
>150	5	5	5	5	5	5

Con el fin de simular la interferencia por lipemia, se enriqueció gradualmente las muestras con la emulsión para perfusión SMOFlipid 200 mg/mL (Fresenius Kabi, Graz, Austria, exp.03/2025), siguiendo los volúmenes de emulsión predeterminados (Tabla 1).

Tras añadir la emulsión lipémica, se repitieron las determinaciones de CBC, y se determinaron las concentraciones de Hb estándar medidas mediante espectrofotometría.

En el protocolo de verificación para una determinación fiable de los parámetros de CBC en muestras de sangre total lipémicas en el AH Sysmex XN-1000 se incluyeron tres métodos diferentes:

(1) Determinación de las muestras lipémicas en el modo de determinación de reticulocitos para obtener los valores de Hb-O y MCHC-O.
(2) Determinación de la concentración de hemoglobina en muestras de plasma lipémicas centrifugadas.

Las muestras lipémicas enriquecidas se centrifugaron a 400 *g* durante 10 minutos. El plasma lipémico se separó cuidadosamente para no alterar las células y se determinaron las concentraciones de Hb estándar en las muestras de plasma lipémicas. Seguidamente, los valores obtenidos en las muestras lipémicas se ajustaron según las determinaciones en plasma mediante la siguiente ecuación:

$${\text{Hb}}_{\text{corregida}}\left(g/L\right)={\text{Hb}}_{\text{muestra}\;\text{lip}é\text{mica}}–\left[{\text{Hb}}_{\text{plasma}\;\text{lip}é\text{mico}}\times \;\left(1\;–\text{ hematocrito }\left(L/L\right)\right)\right]$$

Los parámetros MCH y MCHC se calcularon siguiendo la ecuación:

$$\text{Hb }\left(g/L\right)\text{ Hb }\left(g/L\right)$$

$$\text{MCH}=\left(\text{pg}\right)\text{ MCHC}=\left(g/L\right)$$

$$\text{Erc }\left(\times \;10^{12}/L\right)\text{ Hto }\left(L/L\right)$$

(3) Sustitución del plasma lipémico eliminado por el volumen correspondiente de diluyente del analizador.

El plasma eliminado se sustituyó con el diluyente del analizador (Sysmex Cellpack™ DCL). Tras mezclarlo meticulosamente, se repitieron las determinaciones y se anotaron las concentraciones de Hb estándar.

Para comprobar el grado de lipemia simulado, se determinaron las concentraciones de triglicéridos y el índice HIL en muestras de plasma lipémicas en un analizador Abbott Alinity c (Abbott, Abbott Park, IL, EE.UU.). Los triglicéridos se determinaron mediante el método de glicerol fosfato oxidasa, que genera el colorante de quinoneimina, cuya absorbancia, determinada a 604 nm es proporcional a la concentración de triglicéridos presente en la muestra. Los índices HIL se midieron mediante espetrofotometría con distintos pares de longitudes de onda y un algoritmo específico para obtener valores de índice HIL que aproximadamente se corresponden con las concentraciones de hemoglobina libre, bilirrubina y triglicéridos.

En la Tabla 2 se describe el protocolo completo.

**Tabla 2:** Protocolo de verificación para una determinación fiable de los parámetros de CBC en muestras lipémicas de sangre total con K_2_EDTA en el analizador de hematología Sysmex XN-1000.

Pasos	Análisis	Adicional
Determinación de CBC en muestras nativas no lipémicas	Hemoglobina, la MCH y la MCHC determinadas en muestras nativas se considerarán como “valor real”	Impresión de la medida de CBC original
Adición de la emulsión lipídica	Se añade una emulsión lipídica Smoflipid a las muestras seleccionadas. Se comienza con 20 μL y se va aumentando progresivamente el volumen	Se mezclan las muestras suavemente para distribuir de forma homogénea la emulsión
Determinación de Hb-O y MCHC-O	Se analizan las muestras lipémicas en el modo de medición de reticulocitos	La opción de medida de Hb-O y MCHC-O se encuentra en la pestaña “*lab only*”.
Centrifugación de la muestra lipémica	Las muestras lipémicas se centrifugan a 400 *g* durante 10 minutos	Se selecciona el programa CSF de la centrifugadora
Se elimina el plasma lipémico	Tras la centrifugación, se anota el nivel de plasma de la muestra de CBC	Se separa el plasma lipémico cuidadosamente y se introduce en un tubo limpio de plástico utilizando una pipeta automática
Determinación de la Hb en muestras de plasma lipémicas	El plasma lipémico separado Se analiza utilizando el modo de muestreo abierto del analizador Sysmex XN-1000 analyzer	Se imprime el resultado de Hb en el plasma lipémico. El plasma se envía a Bioquímica para la medida el índice HIL
Adición del diluente del analizador	Se añade el diluyente del analizador (Sysmex Cellpack™ DCL) a la muestra de CBC hasta la marca que indica hasta dónde llegaba el plasma	Se mezcla la muestra suavemente para distribuir de forma homogénea la emulsión en la muestra
Reanálisis de la muestra	Las muestras de CBC se reanalizan en el HA Sysmex XN-1000.	Se imprimen los resultados
Introducción de los resultados	Todos los resultados se introducen en una hoja excel	Se registran todos los posibles problemas que hayan surgido durante la implementación del protocolo

En el presente estudio, no se recogieron datos sobre los pacientes, ya que el estudio de interferencia únicamente incluía muestras de plasma sobrantes con solicitudes de CBC. El Comité de Ética del Hospital Universitario Sveti Duh de Zagreb de Croacia aprobó de manera universal los estudios de verificación en muestras sobrantes y confirmó que no era preciso obtener el consentimiento de los pacientes.

## Análisis estadístico

Aplicando el diagrama de Bland-Altman, se calculó el sesgo medio entre las determinaciones obtenidas con diversos métodos de eliminación de lipemia y las medidas iniciales. El sesgo medio se expresó en valores porcentuales y se comparó con los criterios de variabilidad biológica para el sesgo, según la base de datos de variación biológica de la Federación Europea de Química Clínica y Medicina de Laboratorio (EFLM) [12]. Se realizó la correlación de rangos para evaluar la posible correlación entre los sesgos calculados y el grado de lipemia, así como la concentración de hemoglobina [13]. Dado que las variables no presentaron normalidad en la distribución, las diferencias entre los tres métodos de eliminación de lipemia se analizaron con la prueba de Friedman. Se consideró estadísticamente significativo un valor p<0,05. Todos los análisis estadísticos se realizaron con el paquete MedCalc17.4.4^©^ 23.0.2 (MedCalc Software, Ostend, Bélgica).

## Resultados

### Precisión

Los resultados del estudio de precisión se correspondieron con los criterios de aceptación mostrados en la Tabla 3. Actualmente, la determinación de Hb-O en el AH Sysmex XN-1000 únicamente se emplea en contextos experimentales, sin que el fabricante haya establecido ningún criterio de aceptabilidad de precisión, por lo que se emplearon criterios de variabilidad biológica para la precisión.

**Table 3:** Resultados del estudio de precisión para la determinación de la hemoglobina óptica en el analizador de hematología Sysmex XN-1000.

Precisión, %	Muestras de pacientes									Muestras de control								
	Baja			Media			Alta			L1			L2			L3		
	$\bar{X}$	CV	VB	$\bar{X}$	CV	VB	$\bar{X}$	CV	VB	$\bar{X}$	CV	VB	$\bar{X}$	CV	VB	$\bar{X}$	CV	VB
HB-O	101	1,4	2,0	119	1,6	2,0	141	1,3	2,0	56	1,7	2,0	104	0,9	2,0	132	1,7	2.0

HB-O, medida de hemoglobina óptica en el analizador de hematología Sysmex XN-1000; CV, coeficiente de variación; VB, criterios de aceptación de precisión según la base de datos de variación biológica (mínimo).																		
	Dentro de los criterios de aceptación																	

### Exactitud

Las 15 muestras con mayor grado de lipemia simulada (adición de 120 μL de emulsión lipídica) estaban hemolizadas macroscópicamente y se excluyeron del posterior análisis estadístico. En la Tabla 4 se muestra el incremento progresivo de la concentración de hemoglobina, la medida de MCHC y la concentración de triglicéridos.

**Tabla 4:** Incremento progresivo de la hemoglobina y la concentración MCHC tras la adición de la emulsión lipídica.

Volumen añadido de emulsión lipídica		Hb nativa	Hb+ Emulsión lipídica	Porcentaje de variación	MCHC nativa	MCHC+ Emulsión lipídica	Porcentaje de variación	TG	Índice L	Índice H
20	Mediana	113	117	3,5	336	350	4,1	8,2	264	31
	RIC	97–166	103–170	2,9–5,5	331–341	339–356	3,0–5,8	7,2–9,8	223–387	11–64
40	Mediana	115	124	7,1	333	361	9,1	14,7	497	19
	RIC	97–156	107–162	4,8–9,7	328–339	352–373	6,8–11,7	12,8–18,6	454–622	5–42
60	Mediana	138	148	10,6	331	369	13,1	21,6	803	66
	RIC	90–158	103–173	7,5–14,3	323–340	362–400	9,5–17,8	16,9–30,3	647–1,115	27–92
80	Mediana	127	148	13,9	330	381	15,8	25,7	998	61
	RIC	102–156	117–168	11,2–15,1	318–341	369–399	13,9–18,5	22,8–33,4	797–1,181	16–91
100	Mediana	134	158	17,9	332	404	21,4	40,5	1,539	37
	RIC	94–157	125–176	12,8–27,0	314–341	389–438	13,8–36,3	32,7–45,7	1,192–1712	22–94

CVR (valor de referencia del cambio) de la hemoglobina=6,8 %; CVR de la MCHC=3,0 %										
	Dentro del CVR						Superior al CVR			

En la Tabla 5 se muestran los resultados del estudio de exactitud. Se observó una diferencia no estadísticamente significativa y aceptable entre el valor de Hb-O y de Hb inicial, siguiendo los criterios más estrictos de aceptabilidad (−0,4 %, IC95 %: −1,2–0.3, p=0,2447). La hemoglobina medida en muestras de plasma lipémico reemplazadas por un diluyente del analizador mostró un sesgo mínimo, aunque estadísticamente significativo (−1,1 %, IC95 %:−2,0 – (−0,1), p=0,025). El sesgo mayor e inaceptable se obtuvo al recalcular los valores de hemoglobina a partir de la hemoglobina medida en plasma (−3,5 %, IC95 %: −4,1 – (−2,9), p<0,0001).

**Tabla 5:** Comparación de la hemoglobina, concentración de MCH y MCHC en los métodos de eliminación de la lipemia.

**Método de eliminación de la lipemia**	**%**S**esgo**	**IC95%**	**Valor p**	**Criterios de variabilidad biológica para el sesgo^a^**		
				**Sesgo óptimo**	**Sesgo deseable**	**Sesgo mínimo**

1. Determinación de las muestras lipémicas en el modo de medida de los reticulocitos						
Hb-O	−0,4	−1,2–0,3	0,244	0,8	1,7	2,5
MCH (Hb-O/RBC)	−1,2	/	/	0,6	1,2	1,7
MCHC-O	1,3	/	/	0,2	0,4	0,6
MCHC (Hb-O/HCT)	−0,5	/	/	0,2	0,4	0,6
2. Determinación de la concentración de hemoglobina en muestras de plasma lipémicas centrifugadas						
Hb corregida	−3,5	−4,1 – (−2,9)	<0,001	0,8	1,7	2,5
MCH calculado	−4,4	/	/	0,6	1,2	1,7
MCHC calculado	−1,5	/	/	0,2	0,4	0,6
3. Sustitución del plasma lipémico eliminado con el diluyente del analizador						
Diluyente de Hb	−1,1	−2,0 – (−0,1)	0,025	0,8	1,7	2,5
Diluyente de MCH	2,5	/	/	0,6	1,2	1,7
Diluyente de MCHC	2,7	/	/	0,2	0,4	0,6
MCHC (diluyente de Hb/HCT)	0,7	/	/	0,2	0,4	0,6

^a^Aarsand AK, Fernandez-Calle P, Webster C, Coskun A, Gonzales-Lao E, Diaz-Garzon J, et al. Base de Datos de Variación Biológica de la EFLM. https://biologicalvariation.eu/. Sesgo permitido: < 0,25 × (CVI^2^ + CVG^2^)^1/2^ El factor 0,25 hace referencia a las Especificaciones de Calidad Analítica (APS) El factor de las especificaciones de calidad óptica y mínima son 0,125 y 0,375, respectivamente. CVI - Variación intraindividual estimada (CVI); CVG-variación interindividual estimada (CVG) Disponible en:Aarsand AK, Fernandez-Calle P, Webster C, Coskun A, Gonzales-Lao E, Diaz-Garzon J, et al. Base de Datos de Variación Biológica de la EFLM. https://biologicalvariation.eu/. Hb-O - determinación de hemoglobina óptica en el analizador de hematología Sysmex XN-1000; MCHC-O - determinación de la concentración de MCHC óptica en el HA Sysmex XN-1000; RBC - recuento de glóbulos rojos. HCT- Hematocrito.						
	Cumple criterios de aceptación				Excede los criterios de aceptación	

De acuerdo con los criterios de exactitud establecidos, los resultados de MCHC obtenidos con los tres protocolos de eliminación de lipemia resultaron ser inaceptables. No obstante, si se aplica la corrección matemática al cálculo de MCHC empleando la medida de Hb-O, se obtiene un sesgo aceptable según los requisitos de exactitud mínimamente aceptables, frente al MCHC nativo.

La prueba de Friedman mostró diferencias estadísticamente significativas entre los valores de hemoglobina obtenidos (p<0,00001). Las pruebas post hoc revelaron una diferencia estadísticamente significativa entre la hemoglobina medida en la muestra nativa y la hemoglobina obtenida mediante corrección de la hemoglobina en plasma y la hemoglobina medida en muestras donde se sustituyó el plasma por el diluyente del analizador (p<0,05). No se observaron diferencias estadísticamente significativas entre el valor de hemoglobina inicial y HB-O (Tabla 6). Se obtuvieron los mismos resultados cuando se dividieron todas las determinaciones de hemoglobina por categorías: <110 g/L, 110–150 g/L y >150 g/L, que respectivamente reflejan concentraciones bajas, normales y elevadas de hemoglobina. Las determinaciones de Hb-O fueron igualmente fiables, independientemente de los intervalos de concentración.

**Tabla 6:** Comparación de las medidas mediante la prueba estadística de Friedman para muestras relacionadas.

Comparación de medidasn =75	1. Medida de muestras nativas	2. Medida óptica	3. Corrección de las muestras de plasma lipémicas centrifugadas	4. Sustitución del plasma lipémico eliminado con el diluyente del analizador	Valor p
	Mediana (RIC)	Mediana (RIC)	Mediana (RIC)	Mediana (RIC)	
Hemoglobina (todas las muestras)	132 (101–158)	129 (102–158)	124^a^ (97–151)	126^a^ (98–155)	<0,00001
<110 g/L	92 (83–98)	91 (83–96)	89^a^ (83–96)	92 (86–98)	0,00484
110 – 150 g/L	128 (119–137)	127 (121–137)	122^a^ (116–131)	125^a^ (119–133)	0,00001
>150 g/L	162 (158–167)	163 (158–170)	158^a^ (155–167)	155^a^ (151–164)	<0,00001
MCH	30,2 (28,4–31,2)	30,2 (28,3–31,2)	29,2^a^ (27,5–30,1)	30,9^a^ (29,3–32,6)	<0,00001
MCHC	334 (323–340)	340^a^ (324–353)	331^a^ (319–337)	341^a^ (331–348)	<0,00001

^a^Diferencia estadísticamente significativa *post-hoc* con respecto a la medida inicial en la muestra nativa (p<0,05).

El análisis estadístico de correlación de rangos analizó las posibles correlaciones entre los sesgos observados en la determinación de Hb con los tres métodos de eliminación de lipemia y el grado de lipemia o hemólisis (determinado mediante la concentración de triglicéridos y los índices HIL). En la Tabla 7 se muestran los resultados.1

**Tabla 7:** Correlación entre las desviaciones en la determinación de la hemoglobina en muestras lipémicas y el grado de lipemia y hemólisis.

Sesgo calculado	Triglicéridos, mmol/L		Grado de lipemia (Índice L)		Hemólisis (Índice H)	
	Rho (IC 95 %)	Valor p	Rho (IC 95 %)	Valor p	Rho (IC 95 %)	Valor p
Hb-O	−0,072 (−0,295–0,157)	0,537	−0,041 (-0,266–0,187)	0,725	0,068 (−0,161–0,291)	0,559
Hb corregida	−0,915 (−0,946 – (−0,868))	<0,001	−0,918 (-0,947 – (−0,873))	<0,001	−0,390 (-0,566 – (−0,178))	0,001
Diluyente de Hb	−0,369 (−0,550 – (−0,155))	0,001	−0,412 (-0,590 – (−0,196))	0,001	−0,406 (−0,585 – (−0,190))	0,001

	Correlación estadísticamente significativa.					

## Discusión

La determinación de Hb-O con el analizador Sysmex XN-1000 manejó con eficacia la interferencia por lipemia en muestras de sangre total EDTA lipémicas. Al reanalizar las muestras en el modo de determinación de reticulocitos, se eliminó la necesidad de realizar los procedimientos manuales tradicionalmente empleados en los laboratorios clínicos para eliminar la interferencia por lipemia.

Aunque se observó una elevación significativa de las concentraciones de hemoglobina (Hb), incluso con la adición más pequeña de lípidos (20 µL), dicha elevación no fue clínicamente significativa si se comparaba con los valores de referencia del cambio (RCV) obtenidos de la base de datos de la EFLM. La concentración de triglicéridos en dichas muestras podía llegar a alcanzar los 10 mmol/L. Sin embargo, la adición suplementaria de la emulsión de lípidos provocó que las concentraciones de Hb superaran la significación clínica en los RCV, lo que nos urgió a reconsiderar el punto de corte de Sysmex para MCHC de 365 g/L como indicador de muestra lipémica para el procesamiento del CBC. Con el propósito de minimizar análisis innecesarios y, a su vez, poder identificar la interferencia por lipemia, sugerimos un umbral para el MCHC más bajo, de 360 g/L, que podría mejorar la gestión de interferencias analíticas y resulta similar a las recomendaciones realizadas por Henry y col. [14].

Otra ventaja de la determinación de Hb-O, frente a los protocolos existentes para la eliminación de la lipemia, es su independencia de los niveles de lipemia y hemólisis. Está ampliamente demostrado que niveles elevados de lípidos se asocian a una hemólisis aumentada, tal como también muestra nuestro estudio [15]. El método tradicional para manejar las interferencias por lipemia en las determinaciones de CBC en Croacia, esto es, determinar la concentración de hemoglobina en muestras de plasma lipémicas centrifugadas, se ve afectado significativamente por el grado de lipemia y hemólisis, generando resultados poco fiables en las muestras con niveles elevados de lipemia. Estos resultados coinciden con los obtenidos por Aruga y col., subrayando la efectividad del método óptico a la hora de medir la hemoglobina en muestras quilosas [16]. Sin embargo, en ese estudio no se realizó una comparación con los métodos tradicionales para eliminar la lipemia.

El presente estudio presenta algunas limitaciones. Los resultados podrían variar según el tipo de emulsión de lípidos empleado para simular la interferencia por lipemia. No obstante, esta es la mejor manera de simular la lipemia en los laboratorios. La falta de un control interno de calidad o la no determinación de la Hb-O suponen una inquietud potencial con respecto al uso de este parámetro experimental en los análisis clínicos rutinarios. Aunque la Hb-O mostró una mayor imprecisión total que las concentraciones de Hb medidas mediante espectrofotometría, la imprecisión observada cumplía con los requisitos de aceptación de Sysmex. Las muestras de QC interno de Sysmex se pueden seguir validando para la determinación de Hb-O en diferentes lotes de QC.

A pesar de sus pequeñas limitaciones, este estudio demuestra que la determinación de Hb-O en HA Sysmex es superior a la hora de eliminar la interferencia por lipemia que los métodos de eliminación de lipemia empleados actualmente en los laboratorios de todo el mundo, incluida Croacia. Este es un método sencillo para estandarizar el protocolo de eliminación de la lipemia en las mediciones del recuento sanguíneo completo (CBC) sin precisar la manipulación de las muestras. Las técnicas de eliminación de lipemia suelen implicar la manipulación de la muestra, lo que puede provocar un sesgo significativo, pudiendo derivar en resultados erróneos, y comprometiendo con ello la seguridad del paciente [17], 18]. Finalmente, la adopción de un procedimiento sencillo y seguro para la eliminación de la lipemia mediante la medida directa de la Hb-O podría reducir la carga de trabajo global en los laboratorios, aliviando la ampliamente reconocida escasez de personal [19].
